# Abnormal functional neurocircuitry underpinning emotional processing in fibromyalgia

**DOI:** 10.1007/s00406-023-01578-x

**Published:** 2023-03-24

**Authors:** Thania Balducci, Eduardo A. Garza-Villarreal, Alely Valencia, André Aleman, Marie-José van Tol

**Affiliations:** 1 Postgraduate Studies Division of the School of Medicine, Medical, Dental and Health Sciences Program, National Autonomous University of Mexico, Mexico city, Mexico; 2 Instituto de Neurobiología, Universidad Nacional Autónoma de México Campus Juriquilla, Boulevard Juriquilla 3001, C.P. 76230 Querétaro, QRO Mexico; 3 Instituto Nacional de Salud Pública, Cuernavaca, MOR Mexico; 4 Department of Biomedical Sciences of Cells and Systems, Cognitive Neuroscience Center, University of Groningen, University Medical Center Groningen, Groningen, The Netherlands; 5 Shenzhen Key Laboratory of Affective and Social Neuroscience, Center for Brain Disorders and Cognitive Sciences, Shenzhen University, Shenzhen, China

**Keywords:** Fibromyalgia, Emotion processing, Anterior cingulate cortex, Functional connectivity, fMRI

## Abstract

Fibromyalgia, a condition characterized by chronic pain, is frequently accompanied by emotional disturbances. Here we aimed to study brain activation and functional connectivity (FC) during processing of emotional stimuli in fibromyalgia. Thirty female patients with fibromyalgia and 31 female healthy controls (HC) were included. Psychometric tests were administered to measure alexithymia, affective state, and severity of depressive and anxiety symptoms. Next, participants performed an emotion processing and regulation task during functional magnetic resonance imaging (fMRI). We performed a 2 × 2 ANCOVA to analyze main effects and interactions of the stimuli valence (positive or negative) and group (fibromyalgia or HC) on brain activation. Generalized psychophysiological interaction analysis was used to assess task-dependent FC of brain regions previously associated with emotion processing and fibromyalgia (i.e., hippocampus, amygdala, anterior insula, and pregenual anterior cingulate cortex [pACC]). The left superior lateral occipital cortex showed more activation in fibromyalgia during emotion processing than in HC, irrespective of valence. Moreover, we found an interaction effect (valence x group) in the FC between the left pACC and the precentral and postcentral cortex, and central operculum, and premotor cortex. These results suggest abnormal brain activation and connectivity underlying emotion processing in fibromyalgia, which could help explain the high prevalence of psychopathological symptoms in this condition.

**Supplementary Information:**

The online version contains supplementary material available at 10.1007/s00406-023-01578-x.

## Introduction

Fibromyalgia is a clinical syndrome that mainly affects women [1], characterized by a chronic presence of widespread pain, accompanied by a conglomerate of physical and psychological symptoms. These include fatigue, sleep disturbances, and cognitive dysfunction [2]. Furthermore, depression and anxiety are common comorbidities, with a lifetime prevalence of depression estimated to be higher than 50%, and up to 33% for anxiety [3]. A common finding in fibromyalgia is an imbalance in the presence of positive and negative affect, meaning that patients experience positive affect less frequently than controls, while negative affect is more frequent [4, 5]. This imbalance is not only associated with an increase in the prevalence of depression and anxiety [6], it is also related to higher intensity of pain [7, 8], lower cognitive performance [9], and overall higher severity of fibromyalgia [10]. Because of its relevance for symptomatology, understanding and improving affect in fibromyalgia is an important clinical goal.

Disturbances in emotion processing and regulation have been related to affective instability [11, 12] and might help explain the high comorbidity of fibromyalgia with depression and anxiety. It has been described that patients with fibromyalgia experience emotions with higher intensity and arousal than controls [13, 14]. Additionally, fibromyalgia patients use expressive suppression more frequently as an emotion regulation strategy when compared to controls [14, 15]. Expressive suppression is a strategy frequently associated with psychopathology [16], and it is considered to be maladaptive because when compared to reappraisal, it fails to effectively reduce negative affect and results in increased arousal [13, 14, 17], although findings are not fully consistent [18]. Also, difficulties to identify and describe one's own feelings, known as alexithymia, have been shown to be associated with fibromyalgia and impaired emotion regulation [19–21]. Alexithymia has been related to higher impact of the fibromyalgia [22], depression, anxiety, [23, 24] and pain [19, 25], although not all studies report this relationship [26]. In general, emotion processing and regulation disturbances affect emotional state in fibromyalgia patients and are thought to facilitate the development of depression and anxiety.

Recent advances in the understanding of the physiopathology of fibromyalgia can help elucidate the origin of the emotional difficulties that characterize this disorder. One of the current theories describes fibromyalgia as a central sensitization condition [27, 28], which is triggered by neuro-inflammation [29–31]. The brain areas that have been associated with neuro-inflammation encompass the precuneus, posterior cingulate cortex, midcingulate cortex, supramarginal gyrus, superior parietal lobe, frontal operculum, dorsolateral prefrontal cortex, precentral and postcentral gyrus, medial prefrontal cortex, and the superior frontal gyrus, including the supplementary motor area [29, 31]. Interestingly, many of these brain areas are related to emotion processing and regulation. Roughly, the amygdala, hippocampus, insula, and midcingulate cortex are associated with the generation of the primary emotional response and salience detection. The ventrolateral prefrontal cortex has been associated with signaling the need for control and calling for additional regulatory resources [32–34]. Finally, the dorsolateral prefrontal cortex, supplementary motor area, anterior cingulate cortex, superior parietal lobe, and angular gyrus are related to the implementation of regulation strategies, such as cognitive reappraisal and suppression [32, 35, 36]. Thus, multiple brain regions are orchestrated, to execute a set of cognitive functions that underpin the adequate emotion processing and regulation.

A few previous studies have addressed alterations of brain activity during the processing of emotional stimuli in fibromyalgia [13, 37, 38]. Some of these studies relied on electroencephalography, and one functional magnetic resonance imaging (fMRI) study investigated brain activation associated to the influence of emotions on the processing of painful stimuli [39]. In the current study, we aimed to investigate brain activation and functional connectivity (FC) during emotion processing and regulation in fibromyalgia using fMRI. Specifically, we studied the differences between female fibromyalgia patients and healthy controls (HC) in whole-brain activation and in FC of structures in networks associated with emotional processing and regulation. We focused on female participants because of the higher prevalence of fibromyalgia in this group. Furthermore, we investigated whether individual variation in the clinical presentation of fibromyalgia relates to FC, particularly alexithymia, pain, depression, and anxiety owing to the hypothesized inter-relation between these variables mentioned earlier. To this end, we compared fMRI data acquired from fibromyalgia patients and HC during emotional processing and regulation. We hypothesized higher brain activation in fibromyalgia in the prefrontal cortex, pregenual anterior cingulate cortex (pACC), insula, and amygdala, as well as higher FC between the anterior insula, amygdala and cortical areas involved in emotion processing and regulation, with larger abnormalities in those displaying higher alexithymia and pain, regulated by depression and anxiety.

## Materials and methods

### Participants

The current analysis is part of a bigger project from the National Institute of Psychiatry in Mexico (internal Research Committee registry number: IC18080.0) which aims to study clinical and neuroimaging correlates of emotion processing and regulation in fibromyalgia. For the present analysis, we included participants who performed an emotion processing and regulation task (EPRT) during fMRI: 34 fibromyalgia patients and 33 HC (all females). After fMRI quality control (see *fMRI preprocessing* in the Online Resource document), data from 30 patients with fibromyalgia (mean age = 41.8 years; years of education = 15.5) and 31 HC (mean age = 41.2 years; years of education = 16.8) were analyzed. For details on recruitment and selection criteria, see Online Resource. All participants were right-handed and the groups were matched with respect to age and years of education. All participants provided written informed consent. The protocol was approved by Research Ethics Committee of the National Institute of Psychiatry “Ramon de la Fuente Muñiz” in Mexico City.

### Clinical measures

A series of scales and interviews were administered to assure the compliance with inclusion and exclusion criteria, and to characterize the sample. To confirm right laterality, the Edinburgh Handedness Inventory Short Form was administered [40]. All participants with fibromyalgia had previously received the diagnosis by a rheumatologist or an internist. To confirm (or exclude for HC) the diagnosis of fibromyalgia, we used the American College of Rheumatology 2016 criteria [41]. To exclude major psychiatric disorders in the sample or any disorder in HC, as well as to document other psychiatric comorbidities in the fibromyalgia group (such as depression and anxiety), the Mini International Neuropsychiatric Interview-Plus [42] was administered by a trained psychiatrist. To measure severity of depressive and anxiety, we applied the 17-items Hamilton Depression Rating Scale (HAMD) [43, 44] and the Hamilton Anxiety Rating Scale (HAMA) [45]. Other clinical variables were measured using self-rating scales: the tendency to employ cognitive reappraisal or suppression with the Emotional Regulation Questionnaire [12], alexithymia with the Toronto Alexithymia Scale [46, 47], regular affective state in the past week with the Positive and Negative Affect Schedule [48, 49]. Participants filled the Socio-economic levels Questionnaire of the Mexican Association of Market Research and Public Opinion Agencies Questionnaire (AMAI NSE 8 × 7), which is a standardized instrument to measure socio-economic level in Mexican households, and filled a form with sociodemographic data and clinical characteristics of fibromyalgia (i.e., duration and treatment). Finally, the Fibromyalgia Impact Questionnaire [50] and the McGill Pain Questionnaire [51, 52] were administrated to participants in the fibromyalgia group to evaluate the severity of fibromyalgia and the characteristics of pain, respectively.

### Procedure and fMRI task

Evaluations for each participant were conducted over two sessions with a median time interval of 8 days (range 0–20). During the first session, the interviews and scales mentioned were applied. On the second session, participants were trained to perform the EPRT, and next they underwent the MRI session. Depression, anxiety and affect measurements were updated during the second interview when time interval between sessions was longer than eight days.

**Fig. 1:**
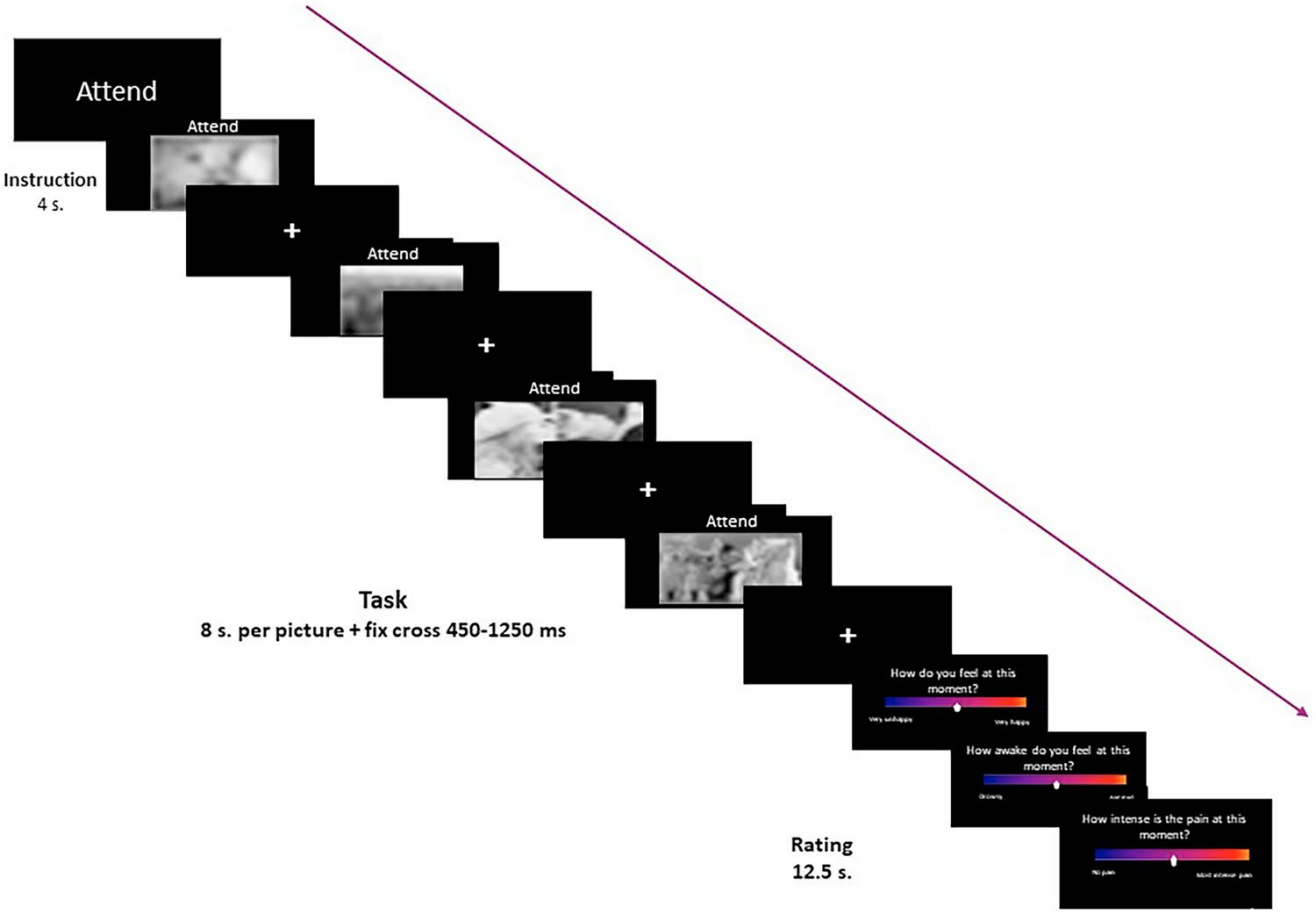
Task design for a single block

For the EPRT, three regulation instructions were implemented: Attend, Reappraise, and Suppress. During the Attend conditions, participants were asked to observe the pictures and allow themselves to experience any emotional response elicited by the stimuli without trying to manipulate the emotional experience. The Reappraise condition had two variants depending on the valence of the stimuli: Increase for positive pictures and Decrease for negative ones. In the case of Increase, participants were asked to boost the positive emotional experience elicited by the stimuli by reinterpreting the presented picture in a more positive self-related manner. In the case of Decrease, participants were asked to reduce the negative emotion elicited by the stimuli by interpreting it as something distant and not dangerous for them or by changing the meaning of the situation to something less negative. For the Suppress conditions (positive and negative), participants were asked to avoid any emotional expression elicited by the stimuli, so that someone watching them would not be able to infer their emotional state. In total, there were seven conditions: Attend neutral, Attend negative, Attend positive, Reappraise negative, Reappraise positive, Suppress negative, and Suppress positive. The task was implemented in a block design (Fig. 1). After each block, participants rated on a visual analog scale the intensity of the emotion elicited, arousal and physical pain. See Online Resource for details on the task.

### fMRI data acquisition

Whole-brain functional and anatomical images were acquired using a 3.0T Philips Ingenia Magnetic Resonance Imaging scanner with a 32-channel phased array head coil. See Online Resource for details on the sequences.

### Clinical and behavioral analyses

Demographic and clinical characterization of the sample was evaluated using non-parametric (Wilcoxon test) and parametric tests. Permutation tests with 1000 iterations were executed for analyzing the behavioral data from the EPRT. Behavioral analyses were done first comparing the ratings (emotional intensity, arousal, and pain intensity) for the combination of all instructions (Regulate, Suppress, and Attend) and valences (neutral, positive, and negative), leading to compare the seven conditions. In consideration of the lack of intra-group effects of conditions on ratings and brain activation (see “Behavioral” and “Whole-brain activation” “Results” sections), we analyzed behavioral data across instructions, leading effectively to comparing the effect of valence (neutral, positive, and negative). To assess the differences between groups in emotion intensity, arousal, and pain, we performed a non-parametric contrast between groups for each variable per task condition (and valence across instructions). Because depression and anxiety may relate to intensity of the emotion, arousal, and pain, we performed Spearman’s correlation between ratings during each valence and the scores of HAMD and HAMA for the fibromyalgia group. Statistics were performed using R 3.6.2 (R Core Team, 2019).

### fMRI preprocessing

The quality of the acquired image sequences was assessed using MRIQC Toolbox [53]. Preprocessing and first-level analysis were executed with SPM12 (Statistical Parametric Mapping, Wellcome Institute for Cognitive Neurology, London, UK) [54]. For details, see the Online Resource.

For the first-level analysis, data was analyzed in the context of the general linear model, with the time series of each participant convolved with the canonical hemodynamic response function and a 128 s high-pass filter applied. The model included the seven conditions (onsets of each picture and durations) plus the instructions, the visual analog scales and, the temporal and dispersion derivatives as regressors. Brain activation during fixation was modeled as implicit baseline.

### Whole-brain activation analysis

Group analyses were performed at the whole-brain level. We planned two repeated measures ANCOVAs: one with the negative conditions (Attend negative, Reappraise negative, and Suppress negative) and one with the positive conditions (Attend positive, Reappraise positive, and Suppress positive) as the within-subject factor. Group (fibromyalgia and HC) was set as the between-subjects factor, and age and education were entered as covariates. Because exploration of the data across all participants revealed lack of significant activations for the main effect of conditions (across groups, and within HC and fibromyalgia separately; even at uncorrected threshold, which was in line with the behavioral results; see “Clinical and behavioral analyses” and “Results”), we set up a 2 × 2 ANCOVA model with valence (positive and negative, across instructions) as the within-subject factor, groups (fibromyalgia and HC) as the between-subjects factor, and age and education as covariates. In this model, the first-level contrasts used were all negative > implicit baseline, and all positive > implicit baseline. This decision allowed us to assess emotion processing and regulation according to the valence of the stimuli, but without differentiating processing from regulation. We modeled the main effect of group, and valence × group interaction. To explore relations of depression and anxiety, the peak activation of the significant clusters was extracted and correlated with the HAMD and HAMA scores.

### Functional connectivity: psychophysiological interaction analysis

The FC was estimated using a generalized Psychophysiological interaction (gPPI) approach. We chose the following seeds (left and right) based on their affiliation with the amygdala–hippocampal network and salience network: hippocampus, amygdala, and anterior insula from the Hammers atlas [55, 56], and pACC five mm spheres were created based on literature about anterior cingulate cortex coordinates relevant for emotion regulation (MNI ± 6 36 10) [36, 57]. In total, eight seeds were defined.

To perform the gPPI analyses, the time series for each seed was extracted and deconvolved to uncover neural activity. Next, the interaction term was created by multiplying the deconvolved seed time series and the task design time series. This interaction term was convolved with the hemodynamic response function to form the gPPI regressor, and the resulting time series was regressed against the rest of the brain to obtain the valence specific FC estimates. Thus, for each seed, a general linear model was estimated in the first-level analysis. In these models, the psychological terms for the gPPI consisted of the contrast between each task condition (i.e., Attend positive) and the implicit baseline, similar to the whole-brain activation analysis model. The other regressors were the time series of the seed and the interaction term. Additionally, temporal and dispersion derivatives were included. Following the across-instruction approach of the main brain activity analyses, the contrasts used in the second-level analysis were: all negative > implicit baseline, and all positive > implicit baseline. To test for the main effects of group and the valence x group effect, we performed an ANCOVA 2 × 2 (valence x group). Next, to test whether depression and anxiety severity moderated the relation between alexithymia, pain, and FC, we extracted the mean connectivity term from the clusters with significant correlation with the clinical variables and performed a partial correlation analysis to control for the contribution of depression and anxiety. The SPM 12 gPPI toolbox was used to perform the gPPI analyses and R for the partial correlations.

### Statistical significance

For all analyses (clinical, behavioral, and fMRI), significant level was set to *p* < 0.05 two-tailed, and False discovery rate (FDR) corrected for multiple comparisons, at a cluster-level for the fMRI data. For the gPPI analysis, an additional Bonferroni correction was applied to account for the number of seeds that were explored.

## Results

### Demographic and clinical characteristics

The demographic characteristics of the 30 fibromyalgia and 31 HC participants are summarized in Table 1. Among the fibromyalgia participants, 56.67% used medication daily (pregabalin being the most prescribed) and 63% fulfilled criteria for at least one current psychiatric disorder, being major depressive disorder the most common (46.7%). For clinical and psychiatric details specific to the fibromyalgia group, see Tables 2S and 3S in the Online Resource. As shown in Table 2, in comparison to HC, fibromyalgia participants presented more pain (*W = 925.5, p* < *0.001*), depressive and anxious symptoms (*W* = *914.0, p* < *0.001*, and *W* = *926.0, p* < *0.001*, respectively), alexithymia (*W* = *720.0 p* < *0.001*), negative affect (*W* = *774.0, p* < *0.001*), and less positive affect (*W* = *190.0, p* < *0.001*). The use of cognitive reappraisal in daily life did not differ between groups (*t(59)* = *− 0.4, p* = *0.73*); for suppression, a non-significant tendency for higher use was found in fibromyalgia (*W* = *590.0, p* = *0.073*).

**Table 1:** Demographic characteristics of the study participants

	FM (*n* = 30)	HC (*n* = 31)	*t/W* value*	*p* value
Age, years (SD)	41.9 (6.3)	41.2 (6.1)	498.5	0.633
Education, *n* (%)				
Elementary	2 (6.7)	1 (3.2)		
High school or technical	9 (30.0)	7 (22.6)		
Bachelor	13 (43.3)	14 (45.2)		
Postgraduate	6 (20.0)	9 (29.0)		
Years of study, years (SD)	15.4 (3.9)	16.8 (4.0)	− 1.361	0.179
Marital status, *n* (%)				0.700
Single	10 (29.4)	7 (21.2)		
Married/cohabitating	17 (50.0)	21 (63.6)		
Divorced/separated	5 (14.7)	4 (12.1)		
Widow	2 (5.9)	1 (3.0)		
Occupation, *n* (%)				
Employed	17 (56.7)	23 (74.2)		0.395
Unemployed/Housewife	11 (36.7)	7 (22.6)		
Student	2 (6.7)	1 (3.2)		
Occupation pattern^†^, *n* (%)				0.088
Full time	10 (47.6)	16 (61.5)		
Half time, fixed schedule	1 (4.8)	5 (19.2)		
Half time, irregular schedule	10 (47.6)	5 (19.2)		
Menstrual cycle phase, *n* (%)^‡^				0.500
Follicular	14 (46.7)	15 (48.5)		
Luteal	8 (26.7)	12 (38.7)		
Postmenopause	4 (13.3)	1 (3.2)		
Unknown^§^	4 (13.3)	3 (9.7)		
BMI, mean (sd)	26.9 (4.2)	24.8 (3.2)	2	0.030
Economic status^¶^, median (range)	C + (AB−D+)	C + (AB−D)	388	0.300

FM, fibromyalgia; HC, healthy controls; BMI, body mass index

^*^W for age and economic status; t for years of study and BMI; Fisher´s Exact Test for marital status, occupation and menstrual cycle phase

^†^Among those who work, FM = 21, HC = 26 participants

^‡^Probable phase according to self-reported last menstrual period date

^§^Six because of hysterectomy, 1 lost data

^¶^The instrument used was the AMAI rule 8 × 7 created for Mexican homes, where A/B is the highest economic status category and E is the lowest

**Table 2:** Clinical characteristics of the study participants

	FM (*n* = 30)	HC (*n* = 31)	F/W/t* value	*p* value	Effect size
VAS for pain during the interview, mean (SD)	45.7 (19.8)	1.2 (3.5)^†^	925.5	< 0.001	0.88
Widespread pain index, mean (SD)	11.8 (4.3)	0.9 (1.4)	922.0	< 0.001	0.86
Symptom severity scale score, mean (SD)	8.3 (2.5)	1.4 (1.5)	904.0	< 0.001	0.82
HAMD total score, mean (SD)	15.2 (6.5)	1.3 (2.0)	914.0	< 0.001	0.84
HAMA total score, mean (SD)	21.2 (7.0)	2.3 (2.5)	926.0	< 0.001	0.85
Positive affect—last week, mean (SD)	25.3 (7.6)	32.5 (4.9)	− 4.0	< 0.001	1.00
Negative affect—last week, mean (SD)	22.9 (8.6)	14.0 (4.4)	774.0	< 0.001	0.57
ERQ reappraisal score, mean (SD)	31.6 (6.6)	32.3 (6.5)	− 0.4	0.729	0.10
ERQ suppression score, mean (SD)	14.7 (6.8)	11.8 (5.0)	590.0	0.073	0.49
TAS difficulty identifying feelings, mean (SD)	23.0 (10.0)	12.0 (6.1)	776.0	< 0.001	0.58
TAS difficulty describing feelings, mean (SD)	14.8 (6.5)	11.0 (4.6)	617.0	0.032	0.28
TAS externally oriented thinking, mean (SD)	20.9 (8.4)	16.6 (5.7)	2.0	0.023	0.60
TAS total score, mean (SD)	58.7 (21.9)	39.5 (12)	720.0	< 0.001	0.47
Alexithymia, *n* (%)				< 0.001	0.46
No alexithymia	14 (46.7)	26 (83.9)			
Possible alexithymia	4 (13.3)	4 (12.9)			
Alexithymia	12 (40.0)	1 (3.2)			

ERQ: Emotion regulation questionnaire, FM, fibromyalgia group; HC, healthy controls; HAMA, Hamilton Anxiety Rating Scale; HAMD, Hamilton Depression Rating Scale; TAS, Toronto Alexithymia Scale; VAS, visual analog scale

*W for VAS for pain during the interview, Widespread pain index, Symptom severity scale, painful tender points (ACR90), HAMD, HAMA, ERQ suppression, TAS total and subscales (except externally oriented thinking), negative effect; Fisher´s Exact Test for alexithymia categories; T for TAS externally oriented thinking, positive effect, ERQ reappraisal

^†^Five healthy controls reported some pain during the interview. This pain was due high physical activity during previous days. Precautions were taken for avoid this kind of pain during the fMRI session

### Behavioral

**Fig. 2:**
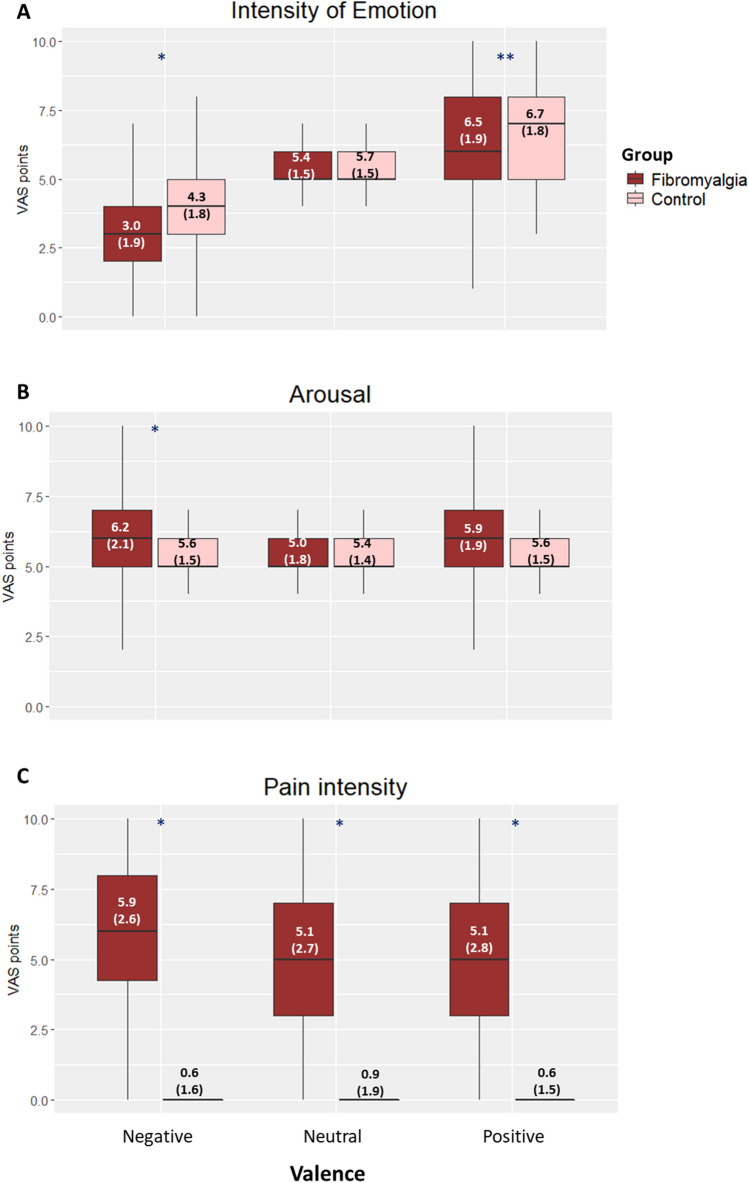
Behavioral results of the emotion regulation task. **A** Intensity of emotion per condition: the visual analog scale (VAS) points represent the valence and intensity of the emotion experienced with 0 corresponds to the most intense negative emotion, 5 is a neutral state and 10 is the most intense positive emotion. **B** Arousal per condition: The visual analog scale (VAS) points represent the arousal with 0 being the minimum level and 10 is the most intense. **C** Pain per condition: the visual analog scale (VAS) points represent the pain intensity with 0 being no pain and 10 is the most intense pain. Mean and SD in the boxes. * Intergroup significant differences (all differences were significant at *p* < 0.001)

We only observed an effect of valence (neutral, positive, and negative), and not of condition (e.g., Reappraise positive, as summarized in Fig. 2 and 1S. Based on this result, we decided to continue the analyses with valence as the independent variable. For effects on pain in fibromyalgia, pain intensity was higher during negative valence compared to neutral (*Z* = *2.31 p* = *0.03*) and positive (*Z* = *3.44 p* = *0.002*) as shown in Fig. 2 panel C; depression (and possibly anxiety) might explain pain during negative valence as these two variables were positively correlated (*r*(26) = 0.43, *p* = 0.02, and *r*(26) = 0.36, *p* = 0.06, respectively). Pain did not differ between valence for HC (mean = 0.8 points, *X*^*2*^*(2)* = *1.0, p* = *0.6)*.

For emotional intensity, fibromyalgia patients experienced emotions more negatively than HC, both for negative (*Z* = *8.0, p* < *0.001*), and positive stimuli (*Z* = 3.0, *p* = 0.009). The arousal was also higher in fibromyalgia for negative stimuli (*Z* = *− 4.0 p* < *0.001*). Depression and anxiety were not correlated to emotional intensity nor arousal in fibromyalgia (Online Resource Table 4S).

### Whole-brain activation

**Fig. 3:**
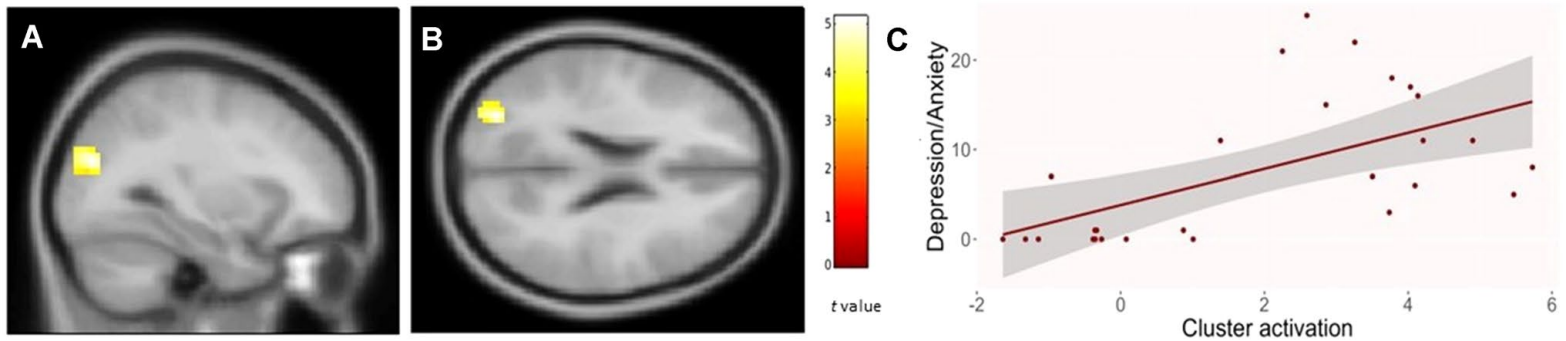
Significant cluster for the fibromyalgia > healthy controls contrast in the whole-brain analysis, located in the left superior lateral occipital cortex (MNI coordinates: − 30 − 79 23), **A** sagittal, **B** axial and **C** correlation of depression/anxiety and cluster activation in FM (*r*(25) = 0.58, *p* = 0.001)

For the 2 × 2 ANCOVA (valence x group) model, we found a significant cluster for the group main effect in the left superior lateral occipital cortex (MNI coordinates: -30 -79 23; F = 26.53, *p* = 0.028). The post hoc *t*-test for the contrast fibromyalgia > HC was significant for that cluster (Fig. 3; *t*(116) = 5.15, *p* = 0.023, *k* = 77). The BOLD activation of this cluster correlated positively with depression (*r*(25) = 0.58, *p* = 0.001), and anxiety (*r*(25) = 0.66, *p* < 0.001) in fibromyalgia.

### Functional connectivity: psychophysiological interactions

**Fig. 4:**
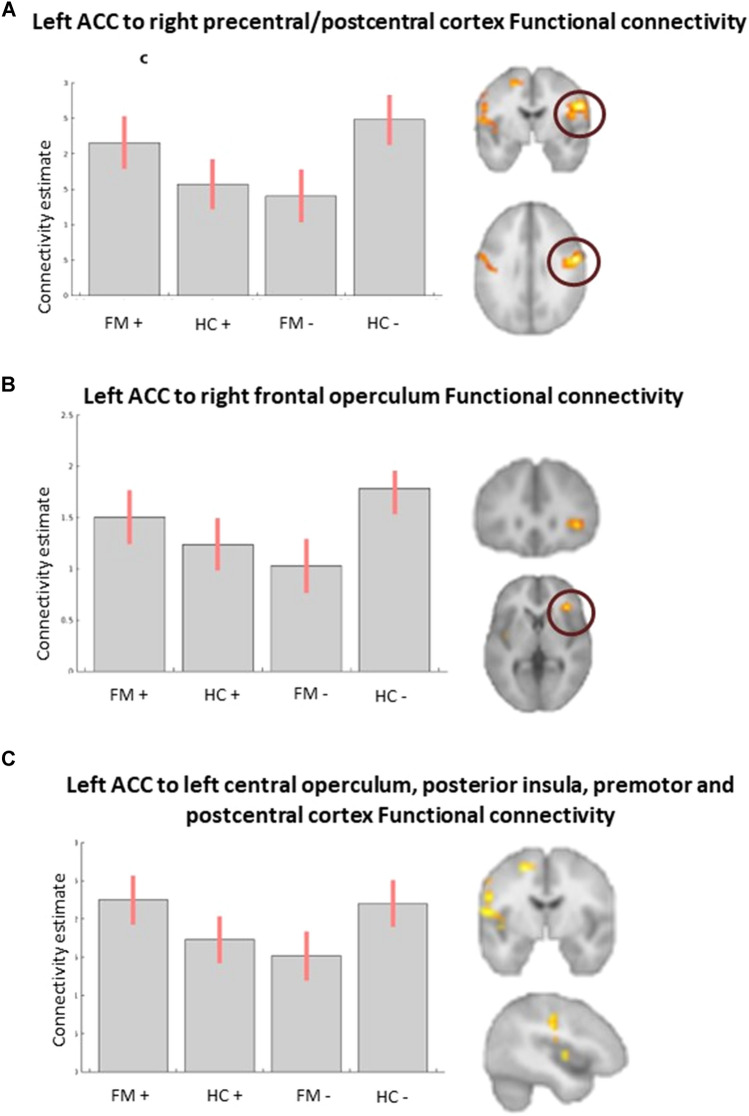
Left ACC task-modulated functional connectivity during emotion regulation. Three clusters were significative for the valence x group interaction effect: **A** right precentral/postcentral cortex, **B** right frontal operculum, and **C** left central operculum, posterior insula, premotor and postcentral cortex. ACC, anterior cingulate cortex; FM, fibromyalgia group; HC, control group; +, regulation of positive valence stimuli; −, regulation of negative valence stimuli

We explored the task-modulated FC of hippocampus, amygdala, anterior insula, and pACC during processing and regulation of emotions. In our analysis, only the left pACC seed showed a valence x group interaction (Table 3). The right post- and precentral cortex, and the left central operculum, premotor and postcentral cortex showed higher FC with the left pACC in fibromyalgia participants than HC during the processing of positive valence stimuli, while during the processing of negative valence stimuli, the FC was lower in these regions (Fig. 4). In fibromyalgia, the FC of left pACC with significant clusters did not correlate with depression nor anxiety (Table 4). The rest of the seeds (bilateral hippocampus, amygdala, anterior insula, and right pACC) did not show significant effects of group nor a valence × group interaction.

**Table 3:** Brain regions per cluster that showed significant task-modulated functional connectivity to the left anterior cingulate cortex during emotion regulation

Region	Size^a^	*F*	*p*	MNI coordinates		
				*x*	*y*	*z*
Valence × group interaction						
R precentral/postcentral cortex	253	33.7	< 0.001	57	− 4	29
L central operculum/premotor/postcentral cortex	284	23.9	< 0.001	− 54	− 13	14

^a^Voxels

**Table 4:** Results of correlation tests between functional connectivity of left anterior cingulate cortex with significant clusters and depression and anxiety in fibromyalgia

Cluster	Stimuli valence	Depression		Anxiety	
		*r*	*p*	*r*	*p*
Valence × group interaction					
R precentral/postcentral cortex	Positive	0.14	0.6	0.18	0.6
	Negative	0.01	1.0	0.09	0.7
L central operculum/premotor/postcentral cortex	Positive	0.30	0.4	0.35	0.4
	Negative	0.12	0.7	0.17	0.6

Finally, a correlation analysis was performed between the FC of each seed and the clinical variables alexithymia and pain in the fibromyalgia group. The *Difficulty to identify feelings* (*DIF*) subscale was the only alexithymia measure that correlated to the FC between right anterior insula and a cluster encompassing parts of the right superior frontal gyrus, the supplementary motor area and the dorsal anterior cingulate cortex (MNI coordinates: *x* = 18, *y* = 5, *z* = 47, size: 43 voxels, *r*(28) = 0.61, *p* = 0.01). Adding depression in a partial correlation moderated the relation between right insula seeded FC and *DIF*, so it was no longer significant (*r*(27) = 0.49, *p* = 0.23). When the correlation analysis was repeated including the control group, no correlation was found. The FC of other seeds did not correlate with alexithymia nor pain.

## Discussion

In this study, we investigated brain activation and FC during emotion processing and regulation in fibromyalgia, using fMRI. We found higher activation of lateral occipital cortex in fibromyalgia during processing of emotional pictures, irrespective of valence. We also found altered valence-dependent FC of the left pACC with the opercular, premotor, post- and precentral cortex during the processing and regulation of emotional stimuli in fibromyalgia. More specifically, we observed higher pACC seeded FC during positive emotion processing and lower FC during negative emotion processing. Our behavioral analysis showed more negative emotions in fibromyalgia than in HC. Taken together, these results suggest that higher negative mood and pain in fibromyalgia are associated with abnormal processing of negative and positive emotional stimuli, which might underpin the emotional difficulties present in fibromyalgia and its effect on pain.

The lateral occipital cortex was found to be hyper-activated in fibromyalgia compared to HC, irrespective of valence, but with variations according to the severity of depression. The lateral occipital cortex has been related to visual attention [58], object perception [59, 60], but also to pain modulation [61]. A recent study found that the decrease in resting-state FC between the occipital cortex and the pACC following transcranial magnetic stimulation relates to a reduction in the affective dimension of pain in fibromyalgia [62], suggesting the occipital cortex has functional relevance for the affective processing of pain. Hypo-activation of visual areas has been already described in other populations with high levels of alexithymia during subliminal emotional stimuli [63]. Here, the observed higher activation of the occipital gyrus during overt emotional stimuli may suggest higher use of visual cues to process and regulate emotions (in opposition to cognitive processes). Additional to pain modulation and alexithymia, emotion processing or depression could explain the differences in occipital activation since this region was found affected in a previous study using a similar emotion processing and regulation task in patients with remitted depression [64], and from our results in the correlation with severity of depression.

Besides the lateral occipital cortex, no other regions showed activation differences, including those from the hypothesis (insula, amygdala, prefrontal and pACC). This could be due to a compensation mediated by changes in the FC as found for the pACC. For the other hypothesized regions, amygdala and prefrontal cortex could be less involved with the emotion processing and regulation difficulties, while the insula alterations seem to depend more on psychological characteristics of the fibromyalgia group, as found in the correlation of FC and clinical variables.

In regard to the pACC, it is an integrative hub associated with the convergence of salient interoceptive, sensitive and emotional stimuli for further processing and coordination of potential motor responses [65]. Furthermore, it is an area rich in opioid receptors [66], thereby participating in the affective assessment of pain [67]. Finally, pACC activation, connectivity, and structure have been implicated in the top-down regulation of negative affect and pain [69–70]. Previously, pACC FC with other structures from a pain inhibitory network was found to be reduced in fibromyalgia [71], and its activation during pain has been related to the availability of μ-opioid receptors and to the affective intensity of pain [72].

We found a hyper-connectivity of pACC during positive stimuli processing and a reduced connectivity during negative stimuli processing with the precentral gyrus, postcentral gyrus, central operculum, and superior frontal gyrus. The precentral and postcentral gyri have been found important for the discrimination of emotions and the representation of valence [73, 74], and to be activated during reappraisal and suppression [32, 75]. It has been reported that these areas are hypoactive in women with premenstrual dysphoric disorder [76], a condition characterized also by emotional dysregulation and pain. The central operculum has been implicated in emotion and pain processing in pathological conditions. For example, in functional dystonia and alcohol dependence, pACC was found hypoactive while performing emotion regulation tasks [77, 78], and in masochist patients, its FC was impaired during a task that involved emotional and painful stimuli [79]. The valence-related alteration of the pACC FC to areas related to interoception and emotion processing and regulation that we found might help explain the relationship between negative affect and higher pain intensity [7, 80], and the deficient pain modulation by positive emotional stimuli [39] in fibromyalgia.

In consideration of the mentioned evidence from the literature and our results, where negative conditions were characterized by higher pain intensity in fibromyalgia and more negative emotional experience (between-groups comparison), we interpret that in fibromyalgia, the processing of emotional stimuli is disrupted according to its valence, given by the affected FC between areas involved in the processing of valence and internal representations encoding. In other words, the pACC FC is affected in fibromyalgia during the processing of emotional stimuli and how it is affected depends on whether the valence of the stimuli is positive or negative.

The involvement of the pACC in linking negative affect and pain is not exclusive from fibromyalgia and might be related to neuro-inflammation. A metabolic marker of neuro-inflammation in the pACC was related to negative affect in patients with chronic low back pain [81]. Although the studies on neuro-inflammation in fibromyalgia [29, 31, 82] have not shown neuro-inflammation this region, neuro-inflammation has been found in areas connected to the pACC such as the midcingulate cortex [83]. Thus, one possibility is that neuro-inflammation affects the pACC through its connection to areas with neuro-inflammation. Alternatively, neuro-inflammation is present in the pACC in fibromyalgia, but it has not been observed due to the employed methodology. Whether neuro-inflammation affects the pACC in fibromyalgia requires further research. In case it is proved, neuro-inflammation could become a treatment target to improve affect and pain modulation in patients with fibromyalgia.

Given the involvement of the anterior insula in emotion processing and regulation [75] and in fibromyalgia symptomatology [84], we studied its FC. We found that in fibromyalgia, during the regulation of negative stimuli, the anterior insula FC to the superior frontal gyrus, supplementary motor area, and dorsal anterior cingulate cortex, areas that are important for emotion regulation, was positively correlated with the *DIF*, a factor of the alexithymia construct. This finding is in accordance to previously described negative correlation of anterior insula activation with alexithymia [85]. Notably, the *DIF* relation to anterior insula connectivity was explained by depression according to our partial correlation analysis results.

### Limitations and future directions

Some limitations need to be taken into account. As for most of the studies in fibromyalgia, our results are limited to women. This is a limitation insofar that we do not know if the findings extend to men. Nevertheless, as a first study, we limited the sample to women to enhance homogeneity, taking into account that the prevalence is considerably larger in women (3.98%, for women vs. 0.01% for men) [1]. Furthermore, participants over 50 years old were excluded to ease task-training, in spite of representativity of elderly patients where the prevalence of fibromyalgia is high [86]. Another limitation pertaining our sample is the presence of physical and mental health comorbidities in the fibromyalgia group with some patients being medicated with drugs that affect central nervous system such as antidepressants. Nevertheless, we required participants to have a stable dose of their medication and to avoid rescue doses 24 h before the brain scan. Although the medication and the mental health disorders could affect the task performance and brain activity, mental comorbidities are highly prevalent among patients with fibromyalgia, which make our findings more generalizable [3, 87], exempting for those using opioids since they were excluded from the study. Furthermore, we performed analyses to control for depression and anxiety.

An important limitation is the lack of effects of condition in the behavioral and the ANCOVA 3 × 2 analyses (condition [reappraise, suppress, attend] x group) in the fMRI analysis. This task has been successfully applied without the suppress condition in a Dutch sample of people with remitted depression [64]. One possible explanation for the lack of clear differences could be that it may have been difficult for our sample to follow the three different regulation instructions (even after training them), as it is suggested by a study using a similar task in a Mexican sample where differences in the effect size of cognitive reappraisal were found according to the procedures to instructing participants [88]. Another possibility is that it may be more difficult for people with fibromyalgia to adequately perform the different emotion regulation strategies than for people with remitted depression. Furthermore, our fMRI results must be taken cautiously, especially the whole-brain activation analysis, since the multiple comparison correction approach was relatively liberal (*p* < 0.05 FDR). In regard to the FC analysis, an additional correction to account for the number of seeds was applied. Finally, our interpretations concerning pain intensity were conceived based on the behavioral results of the task. However, to assess how emotion regulation affects the neural underpinnings of pain, tasks that include painful stimuli along with emotional ones should be applied.

## Conclusions

Taken together, our findings suggest that difficulties in emotion processing and regulation in fibromyalgia are related to alterations in activation of lateral occipital cortex, and in FC of the pACC, a central hub for emotion and pain processing, to other important emotion processing and regulation areas. These alterations might imply that a stronger involvement of regions associated with pain modulation is needed to process emotional stimuli (including valence and pain) in fibromyalgia. As standardized treatment and pharmacotherapy in fibromyalgia are often unsuccessful [89], psychotherapeutic strategies are being used more frequently to treat pain as well as the emotional difficulties and psychiatric comorbidities. Some of these therapies emphasize emotional awareness and regulation [15, 90, 91]. Other therapeutic strategies include neuromodulation techniques [92, 93]. Longitudinal studies are needed to better understand the neural mechanisms of improvement with those therapies. The findings of the current study highlight some of the brain structures for which activation or connectivity might change after treatment.

### Supplementary Information

Below is the link to the electronic supplementary material.

Supplementary file1 (DOCX 293 kb)

## Data Availability

The dataset is described in Scientific Data [94] and publicly available.
