# Supplementary material for: Abnormal functional neurocircuitry underpinning emotional processing in fibromyalgia
Source: Eur Arch Psychiatry Clin Neurosci. 2023 Mar 24;274(1):151–64. doi: 10.1007/s00406-023-01578-x (PMC10786973; doi:10.1007/s00406-023-01578-x)
Supplement: Supplementary file 1 — Supplementary file1 (DOCX 293 kb) [file 406_2023_1578_MOESM1_ESM.docx]

**SUPPLEMENTARY INFORMATION**

**Abnormal functional neurocircuitry underpinning emotional processing in fibromyalgia**

Thania Balducci^1^, Eduardo A. Garza-Villarreal^2^, Alely Valencia^3^, André Aleman^4, 5^, Marie-José van Tol^4^

1 Postgraduate Studies Division of the School of Medicine, Medical, Dental and Health Sciences Program, National Autonomous University of Mexico, Mexico

2 Instituto de Neurobiología, Universidad Nacional Autónoma de México campus Juriquilla, Querétaro, Querétaro, México

3 Instituto Nacional de Salud Pública, Cuernavaca, Morelos, Mexico

4 University of Groningen, Department of Biomedical Sciences of Cells and Systems, Cognitive Neuroscience Center, University Medical Center Groningen, Groningen, the Netherlands

5 Shenzhen Key Laboratory of Affective and Social Neuroscience, Center for Brain Disorders and Cognitive Sciences, Shenzhen University, Shenzhen, China

**METHODS**

***Participants***

Participants were recruited through: (1) educational talks about fibromyalgia at the National Institute of Psychiatry in Mexico City (these talks were open to the general public, therefore, participants from this source included fibromyalgia women with and without psychiatric comorbidity). (2) Patients from the Mexican Foundation for Fibromyalgia. (3) Posters and social media advertisements.

Participants were eligible if they were between 18 and 50 years old, and if they had completed at least elementary school. Fibromyalgia participants needed to be able to stop taking analgesic or benzodiazepine (“rescue-doses”) for at least 24 hours before the MRI-session. Fibromyalgia participants were excluded in case of presence of a major psychiatric disorder (i.e., psychosis, bipolar disorder, obsessive compulsive disorder as screened with the Mini International Neuropsychiatric Interview-Plus), neurological illness, cardiovascular disease, when the pain generated by other conditions was higher than the pain generated by fibromyalgia, and use of opioids. Exclusion criteria for controls were: any acute or chronic pain, any psychiatric disorder, any neurologic-, autoimmune-, cardiovascular-, inflammatory- or rheumatology illness. Additional exclusion criteria for all participants were: more than three sessions of psychotherapy in the last 12 months or any other intervention that could influence emotional regulation (e.g., mindfulness, yoga, counseling); perimenopause, defined as irregular menstrual cycles when previously regular, or less than a year after last menstrual cycle; and any MRI-contraindications.

***Emotion processing and regulation task***

The task was implemented in a block design (Fig. 1). Each condition was repeated three times. Each repetition of a condition was defined as a block. Each block contained the instruction for that block (Attend, *Increase*, *Decrease* or Suppress), followed by four pictures, and three evaluation screens with a visual analogue scale on each. Using the visual analogue scales participants rated the intensity and valence of their current affective state (“How do you feel at this moment”), the arousal (How awake do you feel at this moment”), and the physical pain at that moment (“How intense is the pain at this moment?”). Blocks were presented pseudorandomized (first a neutral block, then six emotional blocks in random order, then seven blocks including both emotional and neutral blocks in random order, then six emotional blocks in random order, and finally a neutral block). Blocks were separated by a fixation cross of varying length (8000 to 14500 ms).

The total task duration was approximately 25 minutes. Pictures were selected from the International Affective Picture Set [1]. Pairs with similar content were formed and matched on valence and arousal, as well as to activity and complexity of the scene presented. Of each pair, one picture was used for the regulate conditions and one was used for the attend conditions. Because suppression was not included in the original task, twelve additional pairs of pictures were added using the same criteria (valence, arousal and complexity) as the original set of pairs. Most pictures displayed scenes with humans. The task was implemented using the E-Prime 3.0.3 software [2].

***Task training***

Before the MRI scan, participants underwent a brief training outside the MRI to perform the emotional processing and regulation task. First, they were trained to follow the instructions by using a set of pictures taken from the International Affective Picture System that were not part of the task. The training was finished when participants were able to give examples of the implementation of the task instructions. Next, using another set of pictures, the participants were trained to perform the task within time constrains and to answer the visual analogue scales using a laptop. An additional training to use the response pad (LS-PAIR, Lumina by Cedrus Corp.) occurred in the MRI scanner, just before the task-sequence. Before and after the scanning, participants rated their fatigue on a visual analogue scale; after the scanning, they rated their performance (ability to execute the instruction) for each task condition on a visual analogue scale. Participants received financial remuneration for their time.

***fMRI data acquisition***

The MRI session consisted in a high-resolution 3D T1- weighted sequence, a T2- and two T2*-weighted sequences, one in resting state and one while performing the emotion processing and regulation task. In this study we only used the T1- and the T2* task -weighted images.

The characteristics of the high-resolution 3D T1- weighted images used for registration were: 180 sagittal sections, repetition time = 7000 ms, echo time = 3500 ms, flip angle = 8°, slice thickness = 1.0 mm, acquisition matrix = 240 x 240 and voxel size = 1.0 x 1.0 x 1.0 mm^3^.

The T2*-weighted echo planar images taken during the task were 36 axial (interleaved) slices, with repetition time = 2000 ms, echo time = 30 ms, flip angle = 75°, field of view = 240 mm, slice thickness = 3.0 mm, acquisition matrix = 80 x 78 and voxel size = 3.0 x 3.0 x 3.0 mm^3^.

***fMRI preprocessing***

For quality control, performed using MRIQC Toolbox, framewise displacement threshold was set at 0.9 mm and sequences that exceeded that threshold more than 30% of time points, were excluded from the analysis [3].

Prior to execute the preprocessing pipeline, functional and anatomical images were manually reoriented to the anterior-posterior commissure plane. Preprocessing included slice time correction, realignment, co-registration of the functional images to the anatomical images, normalization to Montreal Neurological Institute (MNI) space and smoothing with a full-width at half-maximum Gaussian kernel of 6 mm. These steps were performed using SPM12 (Statistical Parametric Mapping, Wellcome Institute for Cognitive Neurology, London, UK) [4].

Because of a spin-history artifact observed in the raw data, independent component analysis (ICA) was performed for functional images to remove the artifact and other sources of noise. The ICA was executed at the subject-level extracting 100 independent components using the Infomax algorithm, and ICASSO to ensure the stability of components. One researcher (T.B.) selected the components for next analysis manually. The selection of components for 10% of the sample was done by two researchers to test the validity of the selection (T.B., M.J.vT.). The Group ICA of fMRI Toolbox (GIFT v4.0b, MIALab, University of New Mexico, USA) software was used for ICA.

***Analysis***

***Task construction: behavioral analysis***

The task we used has been applied elsewhere before with valid results [5]. As we added an additional regulation instruction (Suppress) we decided to analyze the balance between blocks in terms of valence and arousal. For this, comparisons were performed across the repetitions of the conditions of the task using non-parametrical tests.

***Effect of time on pain intensity***

We analyzed the intensity of pain according to the task conditions with the idea of investigating whether emotional conditions would affect the pain intensity in the fibromyalgia group. Nevertheless, there could have been other factors influencing pain. For example, we need to consider the cognitive load (which is a product of time) of the task performed by participants whose disorder is characterized by cognitive failures. Following this, we expected a higher pain intensity during the last repetition of conditions of the task in the fibromyalgia group. For this analysis, we performed a within-group across conditions repetitions of pain intensity using non-parametrical tests.

***Task validation: brain activation analysis***

Due to the lack of main effects of conditions (Attend, Reappraise, Suppress) in our ANCOVA 3 x 2 analysis of brain activation, we looked for the main effect of Reappraise negative against fixation cross for healthy controls (HC) with age and education as covariates. We expected to find involvement of the prefrontal cortex.

**RESULTS**

***Task construction***

The within-group analysis of the intensity-valence of the emotion per repetition of conditions (one comparison done for fibromyalgia and one for HC) showed no differences in any group (fibromyalgia: *X^2^(2) = 4.0, p = 0.2*; HC: *X^2^(2) = 3.0, p = 0.3*). In the case of arousal, HC showed no differences (*X^2^(2) = 2.0, p = 0.3)*, while in fibromyalgia the arousal was higher in the first repetition of conditions compared to the third one (6.20 (1.7) vs. 5.54 (2.2) mean (SD) points, *X^2^(2) = 8.0, p = 0.02)*.

***Effect of time on pain intensity***

Pain was no different between repetition of conditions in HC (*X^2^(2) = 5.0, p = 0.08)*, while in fibromyalgia, it was lower in the first repetition of conditions in comparison to the second and third repetitions (mean (SD) per repetition: first 5.0 (2.6), second 5.52 (2.7), third 5.9 (2.8); *X^2^(2) = 12.0, p = 0.002).*

***Task validation: brain activation analysis***

As a main effect of Reappraise negative in HC, we found five significant clusters that comprised the occipital pole, the inferior frontal gyrus, precentral gyrus, angular gyrus, and the posterior cingulate cortex. For details see Table 1S.

| **Table 1S.** Brain regions per cluster that showed significant activation during Reappraise negative in healthy controls. | | | | | | |
| --- | --- | --- | --- | --- | --- | --- |
|  |  |  |  | **MNI coordinates** | | |
| **Region** | **Size*** | ***Z*** | ***p*** | ***x*** | ***y*** | ***z*** |
| *Main effect Reappraise negative* |  |  |  |  |  |  |
| R occipital pole | 113 350 | Inf. | < 0.001 | 12 | -91 | 2 |
| R inferior frontal gyrus / precentral gyrus | 776 | 6.90 | < 0.001 | 54 | 35 | 17 |
| R angular gyrus / lateral occipital gyrus | 220 | 5.75 | < 0.001 | 48 | -46 | 56 |
| R posterior cingulate cortex | 137 | 5.27 | < 0.001 | 3 | -25 | 29 |
| R precuneus | 243 | 5.14 | < 0.001 | 12 | -67 | 41 |
| *Voxels | | | | | | |

| **Table 2S**. Fibromyalgia participants characteristics | |
| --- | --- |
|  | FM  (n = 30) |
| Years with FM diagnosis, mean (SD) | 4.0 (4.6) |
| Years with FM symptoms, mean (SD) | 8.0 (10.2) |
| Pharmacologically treated, n (%) |  |
| Not treated | 3 (10.0) |
| Only crisis medication | 10 (33.3) |
| Daily medication | 6 (20.0) |
| Daily plus crisis medication | 11 (36.7) |
| Number of daily prescribed drugs, n (%) |  |
| None | 11 (36.7) |
| One | 10 (33.3) |
| Two | 3 (10.0) |
| Three | 4 (13.3) |
| Four | 2 (6.67) |
| Mean (SD) | 1.2 (1.27) |
| Type of daily drugs prescribed, n (%) |  |
| Glutamate channel blocker | 13 (43.3) |
| Glutamate channel blocker | 7 (23.33) |
| Analgesics | 5 (16.7) |
| GABA positive allosteric modulator | 5 (16.7) |
| Serotonin reuptake inhibitor | 3 (10.0) |
| Histamine and serotonin antagonist | 2 (6.7) |
| Noradrenaline reuptake inhibitor and presynaptic receptor antagonist | 1 (3.3) |
| Medical comorbidities, n (%) |  |
| Disc herniation | 8 (26.7) |
| Inflammatory bowel syndrome | 3 (10.0) |
| Radiculopathy | 1 (3.3) |
| Uterine myomatosis | 1 (3.3) |
| Any comorbidity, n (%) | 13 (43.3) |
| FIQ, mean (SD) | 33.1 (9.9) |
| FM: Fibromyalgia, FIQ: Fibromyalgia Impact Questionnaire | |
|  | |

| **Table 3S**. Psychiatric comorbidity in FM participants | |
| --- | --- |
| Disorder | N (%) |
|  |  |
| Current disorders |  |
| Major depressive disorder* | 14 (46.7) |
| Dysthymia | 4 (13.3) |
| Agoraphobia | 1 (3.3) |
| Social phobia | 2 (6.7) |
| Specific phobia | 4 (13.3) |
| General anxiety disorder | 3 (10.0) |
| Somatization disorder | 1 (3.3) |
| Body dysmorphic disorder | 1 (3.3) |
| Pain disorder associated with psychological and medical factors | 6 (20.0) |
| Probable premenstrual disorder | 15 (50.0) |
| Past disorders |  |
| Major depressive disorder* | 17 (56.7) |
| Dysthymia | 1 (3.3) |
| Somatization disorder | 2 (6.7) |
| Infancy attention deficit hyperactivity disorder | 1 (3.3) |
| Number of current disorders |  |
| 0 | 6 (20.0) |
| 1 | 10 (33.3) |
| 2 | 7 (23.3) |
| 3 | 5 (16.7) |
| 4 | 1 (3.3) |
| 6 | 1 (3.3) |
| Number of past disorders |  |
| 0 | 12 (40.0) |
| 1 | 16 (53.3) |
| 2 | 1 (3.3) |
| 3 | 1 (3.3) |
| Total of current disorders, median (rank) | 1 (0 – 6) |
| Total of past disorders, median (rank) | 1 (0 – 3) |
| * Includes depressive disorder due to medical disease | |

| **Table 4S.** Correlations between depression (Hamilton Depression Rating Scale score) and anxiety (Hamilton Depression Rating Scale score), and the behavioural variables (emotional intensity, arousal, pain intensity) of the emotion processing and regulation task per condition valence in the fibromyalgia group. | | | | | | | |
| --- | --- | --- | --- | --- | --- | --- | --- |
|  |  | | Depression | | | Anxiety | |
| Behavioural variable | *df* | *r* | | *p* | *r* | | *p* |
| Negative valence conditions |  |  | |  |  | |  |
| Emotional intensity | 28 | -0.25 | | 0.2 | -0.04 | | 0.8 |
| Arousal | 26 | 0.0 | | 1 | -0.12 | | 0.5 |
| Pain intensity | 26 | **0.43** | | **0.02** | 0.36 | | 0.06 |
| Positive valence conditions |  |  | |  |  | |  |
| Emotional intensity | 27 | 0.09 | | 0.70 | 0.07 | | 0.70 |
| Arousal | 27 | 0.03 | | 0.90 | -0.06 | | 0.70 |
| Pain intensity | 28 | 0.32 | | 0.09 | 0.29 | | 0.10 |


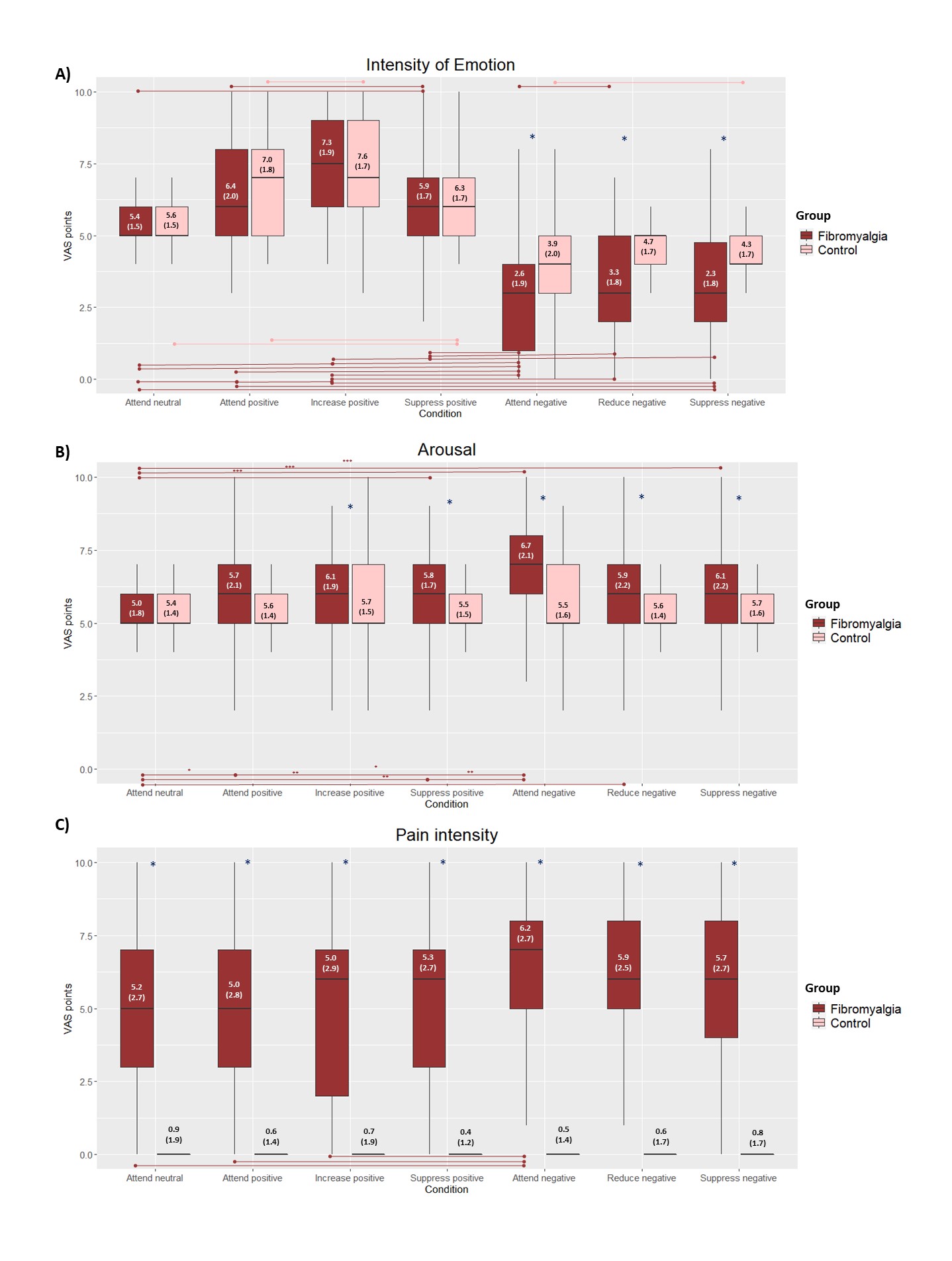


**Figure 1S.** Behavioral results of the emotion regulation task. Horizontal lines show the intragroup comparisons with significant differences and blue asterisk show the intergroup significant differences, mean (SD) in the boxes. A) Intensity of emotion per condition: the visual analogue scale (VAS) points represent the valence and intensity of the emotion experienced with 0 corresponds to the most intense negative emotion, 5 is a neutral state and 10 is the most intense positive emotion. For fibromyalgia, lines above boxes represent contrasts with significant difference p< 0.05, and below boxes represent contrasts with p< 0.001. For controls, contrasts between conditions were significant except for Reduce negative and Suppress negative (*W = 3938.0 p < 0.30*); contrasts lines represent significance level: above boxes p < 0.05, below boxes p< 0.01. For the rest of contrasts with significant difference p < 0.001. B) Arousal per condition: The visual analogue scale (VAS) points represent the arousal with 0 being the minimum level and 10 is the most intense, significance level * p< 0.5, ** p < 0.01, *** p < 0.001. C) Pain per condition: the visual analogue scale (VAS) points represent the pain intensity with 0 being no pain and 10 is the most intense pain. Lines represent the within-group significant differences, significance level p< 0.05.

**REFERENCES**

1. Lang PPJ, Bradley MM, Cuthbert BN (2008) International affective picture system (IAPS): affective ratings of pictures and instruction manual. Technical Report A-8. UNiversity of Florida, Gainsville, FL

2. Psychology Software Tools I (2017) E-prime: Documentation article. In:Psychology Software Tools, Inc.

3. Siegel JS, Power JD, Dubis JW, Vogel AC, Church JA, Schlaggar BL, Petersen SE (2014) Statistical improvements in functional magnetic resonance imaging analyses produced by censoring high-motion data points. Human Brain Mapping 35:1981-1996

4. Friston K (2007) Chapter 2 - statistical parametric mapping. In: Friston K, Ashburner J, Kiebel S, Nichols T, Penny W (eds) Statistical parametric mapping. Academic Press, London, p 10-31

5. van Kleef RS, Marsman J-BC, van Valen E, Bockting CLH, Aleman A, van Tol M-J (2022) Neural basis of positive and negative emotion regulation in remitted depression. NeuroImage: Clinical 34:102988
